# Molecular mechanisms of α-syn abnormal phase separation in cognitive impairment induced by chronic intermittent hypoxia and the neuroprotective effects of Danshensu methyl ester

**DOI:** 10.1186/s10020-025-01366-4

**Published:** 2025-10-29

**Authors:** Juan Li, Na Zhang, Ziyin Zhang, Jinsai Fu, Wenjing Ren, Yi Sun, Shuling Song, Xiaoqian Liu, Jinghui Liu, Jingyu Wang, Yunliang Sun, Kai Zhang, Rongrong Guo, Changjun Lv, Lei Pan, Guiwu Qu, Fang Han, Yan Yu

**Affiliations:** 1 Department of Respiratory Medicine, Binzhou Medical University Hospital, Binzhou Medical University, Binzhou, 256603 China; 2 Department of The First School of Clinical Medicine, Binzhou Medical University, Yantai, 264003 China; 3 School of Basic Medical Sciences, Binzhou Medical University, Yantai, 264003 China; 4 Department of The Second School of Clinical Medicine, Binzhou Medical University, Yantai, 264003 China; 5 School of Pharmacy, Binzhou Medical University, Yantai, 264003 China

**Keywords:** Obstructive sleep apnea, Chronic intermittent hypoxia, Α-synuclein, Reactive oxygen species, Liquid-liquid phase separation, Danshensu methyl ester

## Abstract

The pathological association between α-synuclein (α-syn) deposition and neurodegeneration or cognitive dysfunction has been extensively studied for decades, but significant progress has yet to be achieved in clinical translation. Recently, the introduction of phase separation theory has provided a new perspective for elucidating the molecular mechanisms of α-syn deposition. We made the first discovery that chronic intermittent hypoxia (CIH) exposure induced fear memory deficits by damaging dopaminergic neurons in the ventral tegmental area (VTA). In vivo experiments showed that CIH exposure significantly increased α-syn expression and caused its abnormal deposition in the VTA, leading to neuron dysfunction. Furthermore, inhibiting α-syn expression effectively mitigated neuronal damage and fear memory deficits. Using an intermittent hypoxia (IH) model, we found that abnormal phase separation of α-syn mediates the transition from soluble state to fibrous state, which is the core mechanism of its deposition. α-syn phase separation is regulated by various factors, and we focused on reactive oxygen species (ROS), which were closely associated with α-syn liquid-liquid phase separation (LLPS) and notably altered in CIH/IH conditions. The results showed that CIH-induced ROS promoted the conversion of α-syn-EGFP phase separation droplets to fibrillar state. Moreover, in vitro experiments confirmed that hydrogen peroxide (H₂O₂) treatment induced abnormal phase separation of α-syn, while the antioxidant monomer drug Danshensu methyl ester (DME) effectively inhibited this process. Furthermore, in CIH and IH models, DME intervention significantly reduced ROS levels, inhibited α-syn abnormal phase separation, decreased intraneuronal α-syn deposition, and ultimately improved dopaminergic neuron damage and cognitive function. This study reveals that ROS-regulated abnormal phase separation of α-syn is a novel mechanism underlying CIH-induced neuron damage and might provide insights for the clinical treatment of related cognitive disorders.

## Introduction

As a prevalent sleep-related breathing disorder, Obstructive sleep apnea (OSA) is mainly characterized by nocturnal chronic intermittent hypoxia (CIH) and sleep fragmentation (SF). Establishing an animal model for CIH is a common method to study OSA (Rodriguez et al. 2023). Evidence indicates that CIH can cause irreversible cognitive dysfunction (Skelly et al. 2012; Liu et al. 2020). Previous studies have identified oxidative stress as a significant contributor to cognitive impairment associated with CIH (Zhang et al. 2024; Chen et al. 2005).

Recent research has further implicated the abnormal aggregation of α-synuclein (α-syn) as a critical factor in cognitive dysfunction induced by chronic hypoxia (Li et al. 2022). Additionally, it has been observed that α-syn liquid-liquid phase separation (LLPS) precedes its abnormal aggregation in certain neurodegenerative diseases (Ray et al. 2020). It has been found that many external factors are involved in regulating α-syn LLPS, such as temperature, salt concentration, pH, and reactive oxygen species (ROS) (Mukherjee et al. 2023; Loh et al. 2021). Nevertheless, the precise role of α-syn LLPS and accumulation in neuronal damage and cognitive dysfunction remains to be elucidated in the context of CIH.

Danshensu is a pure molecule, which is derived from *salvia miltiorrhiza*. It has a protective effect on brain diseases due to its excellent antioxidant properties (Tan et al. 2022; Zhao et al. 2008). However, it also presents chemical limitations, such as poor stability, which restricts its clinical application (Wang 2021). Consequently, we synthesized an esterified derivative of Danshensu, Danshensu methyl ester (DME) that possesses a relatively stable chemical structure (Zhu et al. 2022). Our laboratory has demonstrated that DME has better antioxidant function in acute lung injury (Han et al. 2024).

Our work investigated the mechanisms of α-syn LLPS and aggregation and evaluated the consequent neuronal damage in CIH and IH conditions. Moreover, we further explored the effect and underlying mechanisms by which DME attenuates cognitive dysfunction caused by CIH.

## Materials and methods

### Antibodies and reagents

Sensory bacteria (Quanshi Gold, China), anti-alpha-synuclein aggregate (ab209538, Abcam), Alpha Synuclein Aggregate Antibody (2F11, Stressmarq), anti-PSD-95 (ab18258, Abcam), anti-Tyrosine Hydroxylase (AB152, Merck), anti-Synaptophysin (ab32127, Abcam), Alexa Fluor594 Donkey Anti Rabbit Antibody (ab150064, Abcam), Alexa Fluor488 Donkey Anti Rabbit Antibody (ab150073, Abcam), Goat Anti-Rabbit IgG (SA00001-2, Proteintech), β-actin (4970, CST), Danshensu methyl ester (Prepared by Professor Qu Guiwu of our team).

### Animal procedures

64 male wild-type (WT) C57BL/6 mice (6 weeks) were purchased from GemPharmatech Co., Ltd. The mice were kept at a constant temperature of 23 ± 1℃ in a 12-hour light/dark cycle. Standard food and water were supplied. Mice were divided into 4 groups at random: Control group, CIH group, DME group, and DME + CIH group (*n* = 16 per group). Mice in the Control group and DME group were maintained under normoxic conditions. Mice in CIH group and DME + CIH group were exposed to intermittent hypoxia for 10 weeks. During the modeling period, mice in Control group and CIH group were given distilled water by gavage. The mice in DME group and DME + CIH group were treated with DME (10 mg/kg, every other day, at 8:00 PM) by gavage for 10 weeks. Modeling and administration were carried out simultaneously. Each mouse was assigned a distinct identification number, and the animal experiments were sanctioned by the Ethics Committees on Animal Experimentation of Binzhou Medical University (Approval No. 2022 − 202).

### CIH exposure

The CIH model was developed based on previous studies (Silva and Schreihofer 2011). There were four phases with one cycle of 200 s. N_2_ was programmed into the chamber (for 85 to 95 s) to reduce the oxygen concentration from 21% ± 1% (normal) to 7% ± 1% (hypoxia), maintained for 25 to 30 s, O_2_ was then injected to restore the oxygen concentration to 21% ± 1% and maintain it for 30 s until the next CIH cycle. The mice in the CIH group were placed in a hypoxic chamber (BioSpherix OxyClycler A84, USA) 8 h a day (8:00 AM to 4:00 PM) for 10 weeks, while mice in the other groups were placed in a normoxic chamber with an oxygen concentration of 21% ± 1%.

### Cell culture

The SH-SY5Y cell line (SCSP-5014) was purchased from CAS Cell Bank and cultured in MEM/F12 medium supplemented with 10% fetal bovine serum (FBS) in a humidified atmosphere containing 5% CO_2_ at 37℃. The IH group and DME + IH group underwent intermittent hypoxia treatment in a hypoxic chamber for six consecutive days following attaching. The Control group and DME group were maintained in a cell culture incubator with oxygen levels at 21% ± 1%.

### IH exposure

The IH protocol consisted of four phases, with one cycle occurring approximately every 80 min. It involved N_2_ injection to decrease oxygen levels from 21% ± 1–3% ± 1% in 20 min, maintaining it for 20 min, followed by a return to 21% ± 1% for 20 min, and a 20-minute maintenance period in the fourth stage, with CO_2_ levels consistently at 5%. DME (20ug/ml) was introduced to the DME group and DME + IH groups a day before the end of the molding process.

### Fear conditioning test

According to previous studies, the equipment included training compartments and video surveillance systems (ZS Dichuang, Beijing, China) (Shoji 2014). On the first day, after adapting to the black box with jasmine scent for 2 min, the mouse received three times of 30-second sound stimuli (3 kHz, 65 dB) with an interval of 30 s. The end of each sound was accompanied by a foot shock (0.7 mA, lasting 2 s), which was the fear memory formation stage. The next day, the mouse was exposed to the same environment for 5 min without sound or foot shock, and the contextual memory test was performed. On the third day, after acclimating to a blue acrylic box with a lemon odor for 2 min, the mouse was subjected to a cued memory test by receiving 30 s sound (65 dB, 3 kHz) three times (30s intervals), and freezing was recorded during the test.

### Immunohistochemical staining

Paraffin sections were removed, treated with xylene and various concentrations of ethanol, incubated with anti-TH (1:5000) and goat anti-rabbit IgG (1:5000), and then incubated in DAB chromogen solution. Subsequently, sections were stained with hematoxylin, dehydrated, sealed with neutral glue, and observed under microscope.

### Immunofluorescence

Frozen sections of brain tissue were incubated with primary antibodies and secondary antibodies, followed by drops of DAPI. The cell culture medium was withdrawn, the cells were blocked with serum, incubated with primary and secondary antibodies, subjected to dropwise addition of DAPI, and analyzed using EVOS M5000 imaging system (Thermo Fisher, USA). Primary antibodies included anti-α-syn aggregate (1:500), anti-PSD-95 (1:200), anti-TH (1:100), and anti-SYN (1:200). Secondary antibodies included Alexa Fluor 594 Donkey Anti-Rabbit Antibody (1:200) and Alexa Fluor 488 Donkey Anti-Rabbit Antibody (1:200).

### Transmission electron microscopy (TEM)

Fixed the VTA region successively in 3% glutaraldehyde and 1% osmic acid, dehydrated through a gradient of different concentrations of ethanol and acetone, and embedded overnight by immersion in acetone and resin solution. Finally, ultrathin sections at 50 nm were cut on a CM1900 microtome for electron microscopy to obtain the microstructure of the VTA. Randomly selected three mice per group, obtained six electron microscopy images per mouse, and randomly selected areas within each image to statistically analyze the number of synapses, synaptic vesicles, and the area of PSD (Merchan-Pérez ). Neurons in the VTA were visualized using the FD Rapid GolgiStain™ Kit (FD Neurotechnologies, Inc., USA).

### Cell transfection

After cell preparation, 50 µL Opti-MEM was added to 1.0 µL Lipofectamine TM2000, mixed, and left for 10 min. The transfection reagent was subsequently mixed with the siSNCA diluent and incubated. 100 µL of transfection complex was added to 24-well plates and cell cultures were performed. Five hours after transfection, the cell culture medium was replaced. The effect of siRNA silencing was measured 72 h later. The siNC (small interfering negative control) was used as a negative control group.

### Western blotting

BCA assay (Solarbio, China) was used to detect the protein concentration. Primary antibodies included anti-α-syn aggregate (1:1000), anti-PSD-95 (1:1000), anti-TH (1:1000), anti-SYN (1:1000), and β-actin (1:1000). Secondary antibodies included Goat Anti-Rabbit IgG (1:5000). The samples were then developed in a chemiluminescence imaging system.

### Expression and purification of Recombinant α-syn

The primers were as follows (SNCA, FP: 5′-CGCCCATGGGATGTATTCATGAAAGGAC-3′, RP: 5′-CCGAAGCTTGGCTTCAGGTTCGTAGTC-3′). PCR amplification of α-syn was performed using synthetic upstream and downstream primers. The recombinant plasmid pET-22b-EGFP-SNCA was constructed by EGFP-SNCA and PET-22b vector (p0017, Miaoling Plasmid Platform), which was then transferred into *E. coli* BL21(DE3). The Strain (TransGen Biotech) was cultivated in LB liquid medium at 37℃ until the absorbance (A600) reached an optimal density range (0.8-1.0), and then, they were induced with 1mM IPTG at 37℃ overnight. Recombinant PET-22b-α-syn was expressed and purified by the Ni column. SDS-PAGE and Coomassie Blue staining were utilized to evaluate purified proteins.

### Electrophysiological detection of porous microelectrode array

SH-SY5Y cells were induced to differentiate by Retinoic Acid (RA) and 12-O-Tetradecanoylphorbol-13-acetate (TPA), which were first spread inside the microelectrode plate, and then the cells were placed inside the analysis system at 37 °C, 5% CO_2_, 12.5 kHz. The frequency, number, and amplitude of neuronal discharges were recorded and analyzed in real-time.

### Confocal microscopy observation of phase separation

The purified EGFP-SNCA protein was mixed with 10% PEG8000 and incubated in a glass dish for in vitro studies, and the LLPS phenomenon was observed using a laser confocal microscope. In cell experiments, SH-SY5Y cells were transfected with EGFP-SNCA under normoxic or intermittent hypoxic conditions and LLPS phenomenon was performed.

### Fluorescence recovery after photobleaching (FRAP)

The position of the mixed droplet of purified protein and bleach was found under the objective lens and the parameters were adjusted. The fluorescence intensity at the photobleached site was recorded over time using confocal scanning microscopy. The experimental parameters were set as follows: excitation wavelength of 488 nm, photobleaching duration of 0.9 s, scanning interval of 1.65 s, and total scanning time of 120 s. GraphPad Prism software was used for analysis.

### ROS detection

The ROS content in SH-SY5Y cells was detected by DCFH-DA fluorescent probe (Sigma-Aldrich, USA), and ROS content in VTA region of the mouse midbrain was detected by Oroboros Oxygraph-2 K respirometer.

### Statistical analysis

All statistics are expressed as mean ± standard deviation. All statistical data were analyzed using SPSS 26.0. Single-factor ANOVA and Bonferroni post-hoc test were used to analyze the results of the four-group experiment, and independent sample T test or non-parametric test were used to analyze the results of the two-group experiment. *P* < 0.05 was considered statistically significant.

## Results

### Impairment of fear memory caused by CIH

**Fig. 1:**
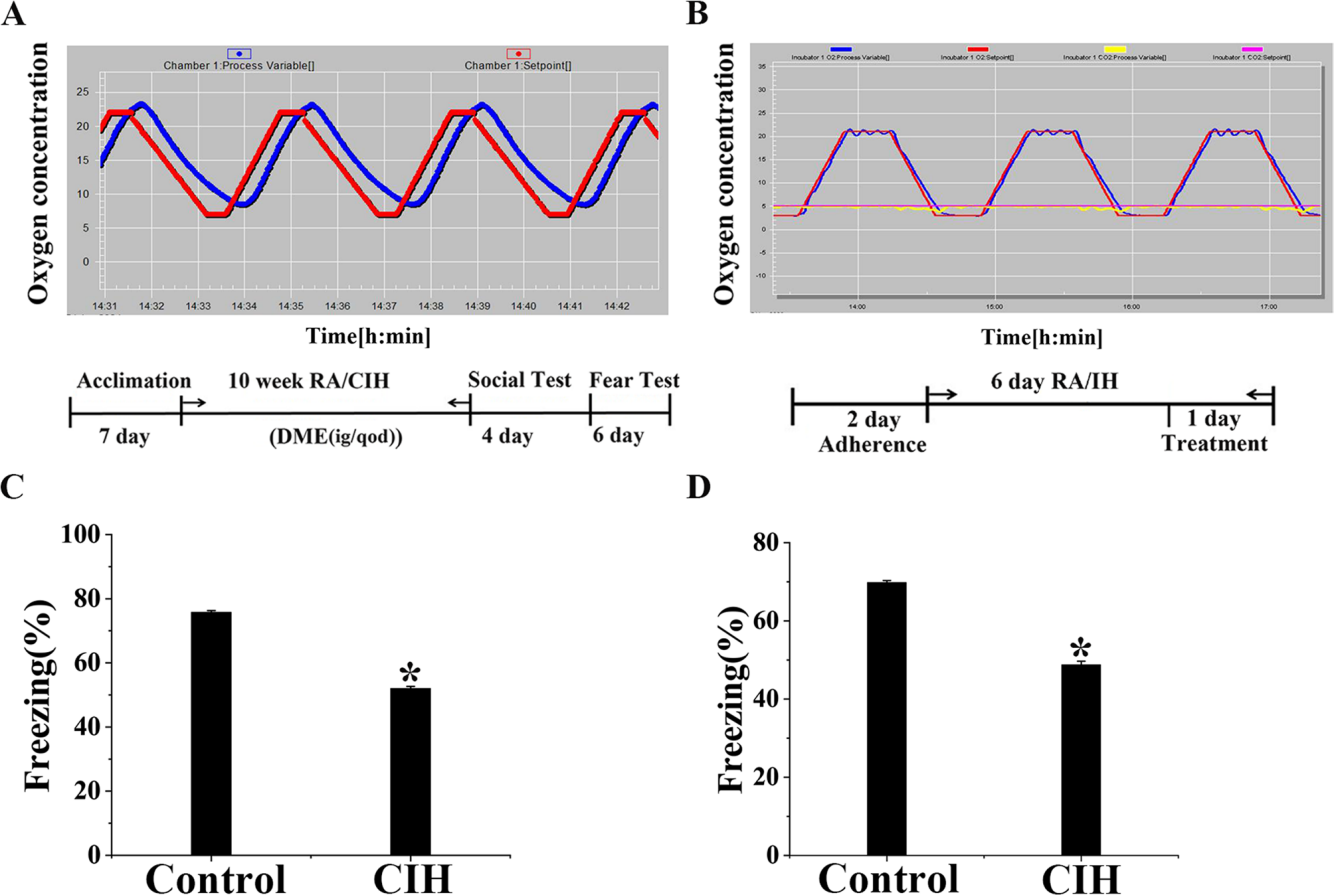
CIH exposure resulted in a significant impairment of fear memory. **A** In the intermittent hypoxia protocol for animals, the red line indicates the preset oxygen concentration, while the blue line shows the actual oxygen concentration. **B** In the IH program, red and blue lines show preset and actual oxygen levels, while pink and yellow lines indicate preset and actual CO_2_ levels. **C** Percentage of freezing in the contextual memory test (*n* = 16). **D** Percentage of freezing in the cued memory test (*n* = 16). Data were presented as mean ± SD. *P < 0.05 vs. Control group

The conditioning fear test was conducted to evaluate contextual and cued memory, aiming to explicitly demonstrate the effect of CIH on the cognitive function of mice. Results showed that, in the contextual memory test, the freezing percentage in the CIH group was approximately 20% lower than in the Control group (Fig. 1C, D, *P* < 0.05). Similarly, the cued memory test exhibited comparable results. These results indicated that CIH exposure impaired contextual and cued memory in mice.

### CIH exposure induced damage of the dopaminergic neurons in the VTA

**Fig. 2:**
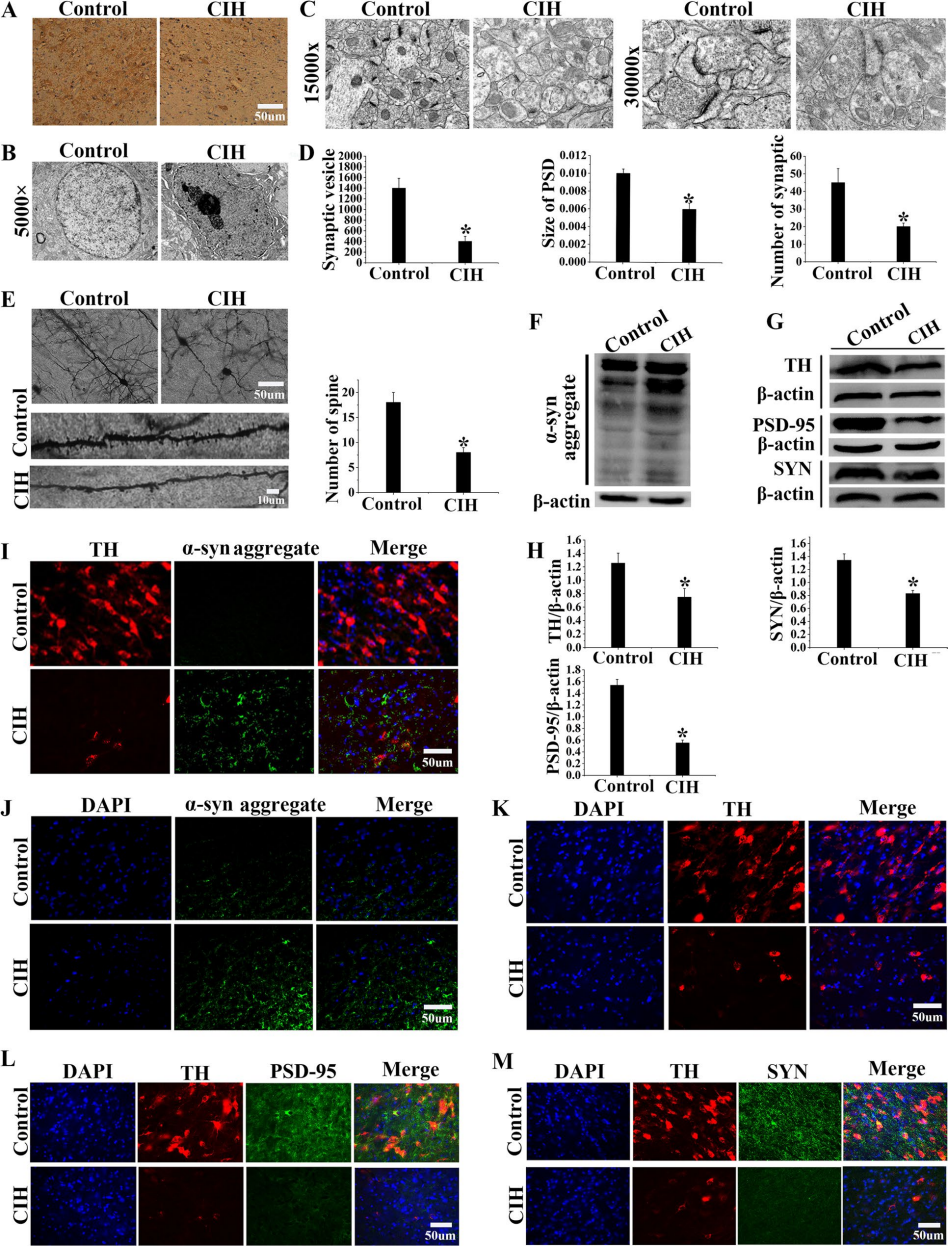
CIH induced the decrease and impairment of dopaminergic neurons in the VTA. **A** Immunohistochemical staining of the VTA showed a decrease in TH-positive neurons in the CIH group. **B** TEM revealed that the nuclei of neurons were deformed, the cytoplasm exhibited vacuolation, lipofuscin accumulation was observed, and organelles were damaged in the CIH group. **C**-**D** TEM and quantification of synapses, synaptic vesicles, and PSD area. **E** Imaging and quantification of Golgi-stained neurons. **F** Western blot analysis revealed an increase in α-syn aggregates within the VTA of the mice. **G**-**H** Western blots and grayscale values of TH, PSD-95, and SYN. **I**-**M** Immunofluorescence staining images of TH, PSD-95, SYN, and α-syn aggregates in the VTA. Data were presented as mean ± SD. *P < 0.05 vs. Control group

Upon completion of the behavioral testing, brain tissues were harvested to probe into the dopaminergic neuronal impairment instigated by CIH. Immunohistochemical analysis showed a decrease in TH-positive neurons in the VTA of CIH-exposed mice (Fig. 2A). In the neurons of the CIH group, TEM revealed cytoplasmic vacuolization, severe nuclear deformation, increased lipofuscin accumulation, and mitochondrial damage (Fig. 2B). Further analysis indicated that the synapses, synaptic vesicles, and PSD area of the CIH group were reduced by approximately 40%, 70%, and 40% (Fig. 2C, D, *P* < 0.05). Moreover, Golgi staining also showed a decreased dendritic spine number, reduced to about half, and lower density in the CIH group (Fig. 2E, *P* < 0.05). Meanwhile, Western blot analysis confirmed decreased TH expression and vital synaptic proteins PSD-95 and SYN, along with increased α-syn expression in the VTA of the CIH group (Fig. 2F-H, *P* < 0.05). The findings were also corroborated by immunofluorescence staining (Fig. 2I-M). The above experiments revealed that CIH exposure caused injury to VTA-DA neurons and might be closely related to the impairment of fear memory.

### The damage observed in SH-SY5Y cells after IH exposure

**Fig. 3:**
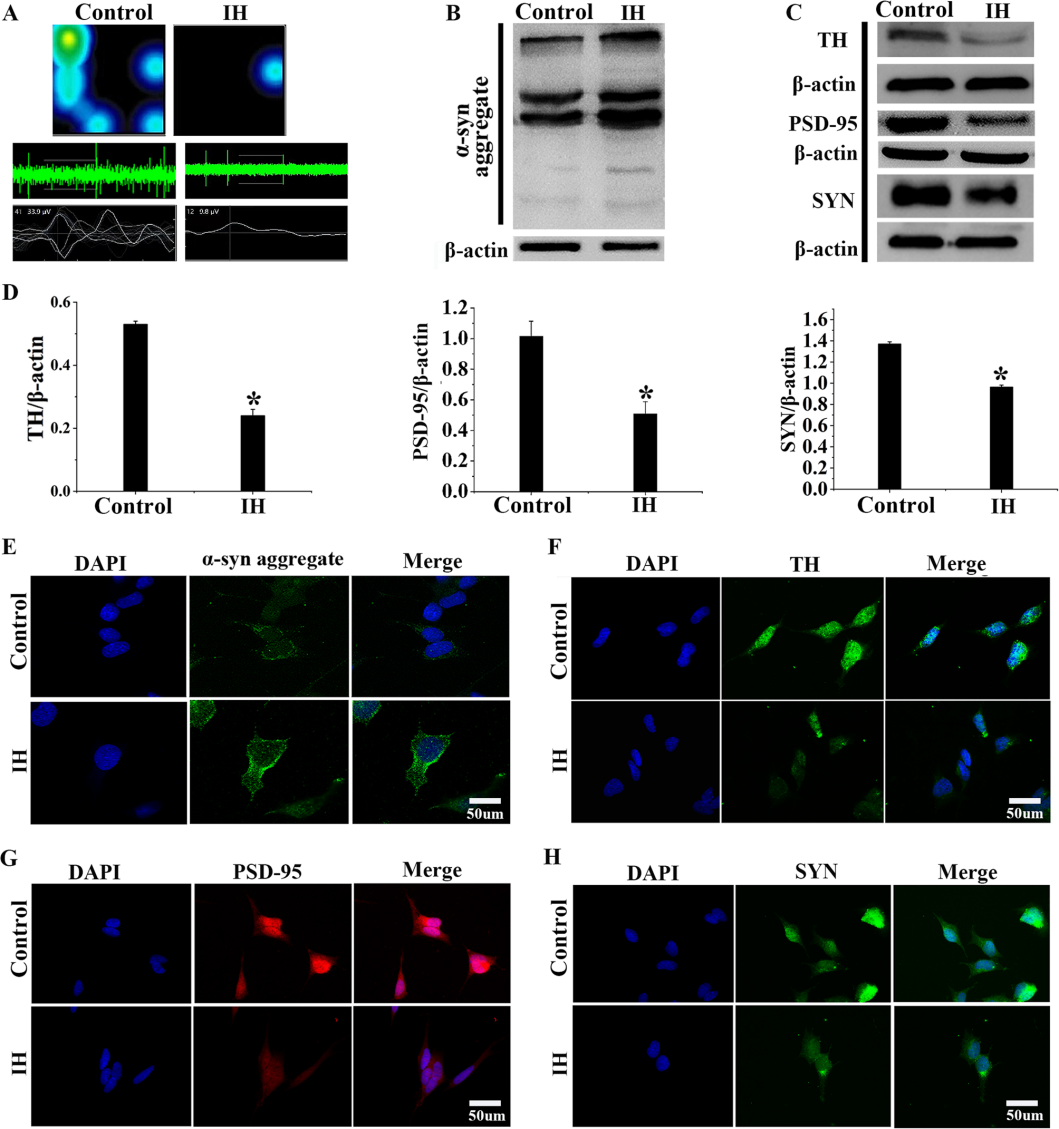
The damage of SH-SY5Y cells by IH**.** **A** MEA results showed that the IH group exhibited downward electrophysiological activity in SH-SY5Y cells. **B** Western blot demonstrated that the aggregation of α-syn elevated in SH-SY5Y cells under IH exposure conditions. **C**-**D** Western blot and gray-value analysis of TH, PSD-95, and SYN. **E**-**H** Immunofluorescence staining images of TH, PSD-95, SYN, and α-syn aggregates in SH-SY5Y cells. Data were presented as mean ± SD. *P < 0.05 vs. Control group

The Microelectrode Array (MEA) showed that SH-SY5Y cells exposed to IH had significantly reduced action potential number, frequency, and amplitude compared to the Control group. This confirmed IH-induced impairment of SH-SY5Y cell function (Fig. 3A). Western blot analysis further verified that expression levels of TH, PSD-95, and SYN in the Control group were approximately 2-fold, 2-fold, and 1.5-fold higher than those in the IH group, respectively. Moreover, a significant increase in α-syn aggregation was observed (Fig. 3B-D, *P* < 0.05). Immunofluorescence staining revealed that, within the IH group, a marked reduction in fluorescence intensity of TH, PSD-95, and SYN occurred, while fluorescence of α-syn was enhanced. (Fig. 3E-H). These findings indicated that IH caused damage to SH-SY5Y cells, particularly synaptic impairment, accompanied by abnormal aggregation of α-syn.

### SiSNCA transfection markedly alleviated SH-SY5Y cell damage after IH exposure

**Fig. 4:**
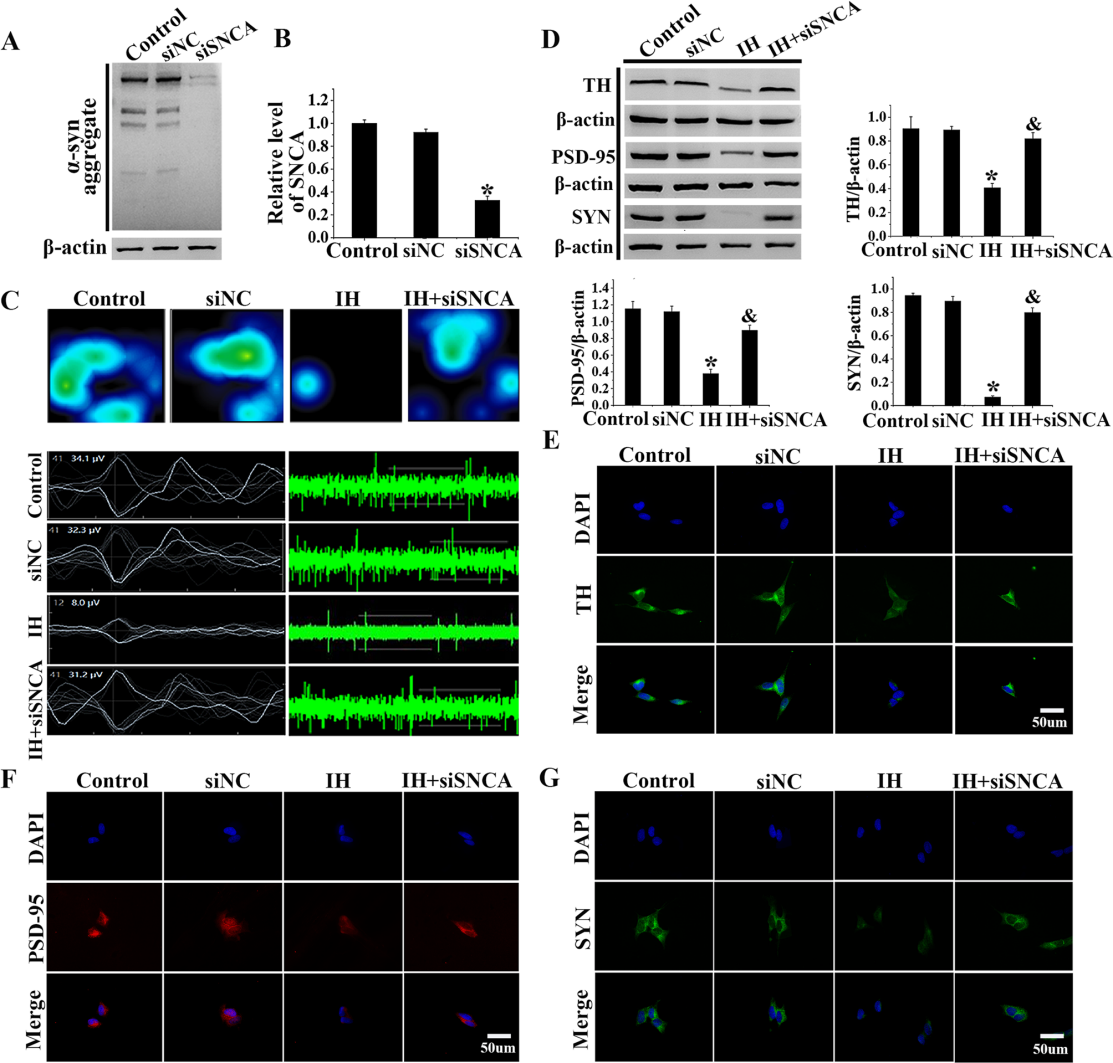
siSNCA transfection markedly ameliorated SH-SY5Y cell damage induced by IH. **A** The efficiency of SNCA knockdown was assessed using Western blot analysis in SH-SY5Y cells. **B** qRT-PCR detected SNCA knockdown efficiency in SH-SY5Y cells. **C** MEA showed that siSNCA effectively inhibited α-syn expression and reduced electrophysiological deficits caused by SH-SY5Y cells injury. **D** Western blot and gray-value analysis of TH, PSD-95, and SYN. **E**-**G** Immunofluorescence staining images of TH, PSD-95, SYN in SH-SY5Y cells. Data were presented as mean ± SD. *P < 0.05 vs. Control group, ^&^*P* < 0.05 vs. IH group

The aforementioned experiments demonstrated that CIH/IH-induced damage to dopaminergic neurons and SH-SY5Y cells was associated with abnormal aggregation of α-syn. To further elucidate the impact of abnormal α-syn aggregation, we modulated the expression levels of the SNCA gene in SH-SY5Y cells through siSNCA transfection. Western blot analysis confirmed a decrease in α-syn protein levels in siSNCA-transfected cells compared to Control group (Fig. 4A). As evidenced by qRT-PCR, the SNCA mRNA levels in the siSNCA group were reduced to one-third of those in the Control group (Fig. 4B, *P *< 0.05). MEA showed that the number, frequency, and amplitude of action potentials of SH-SY5Y cells in the IH group were significantly reduced. However, this impairment was mitigated in the IH + siSNCA group (Fig. 4C). Western blot and immunofluorescence staining revealed that the IH + siSNCA group had higher levels of TH, PSD-95, and SYN, with increases of approximately 2-fold, 2-fold, and 10-fold, respectively (Fig. 4D-G, *P* < 0.05). These results collectively demonstrated that interfering with α-syn expression by siSNCA alleviated the decrease in electrophysiological activity and improved function in SH-SY5Y cells.

### Characterization of α-syn LLPS in SH-SY5Y cells under IH conditions

**Fig. 5:**
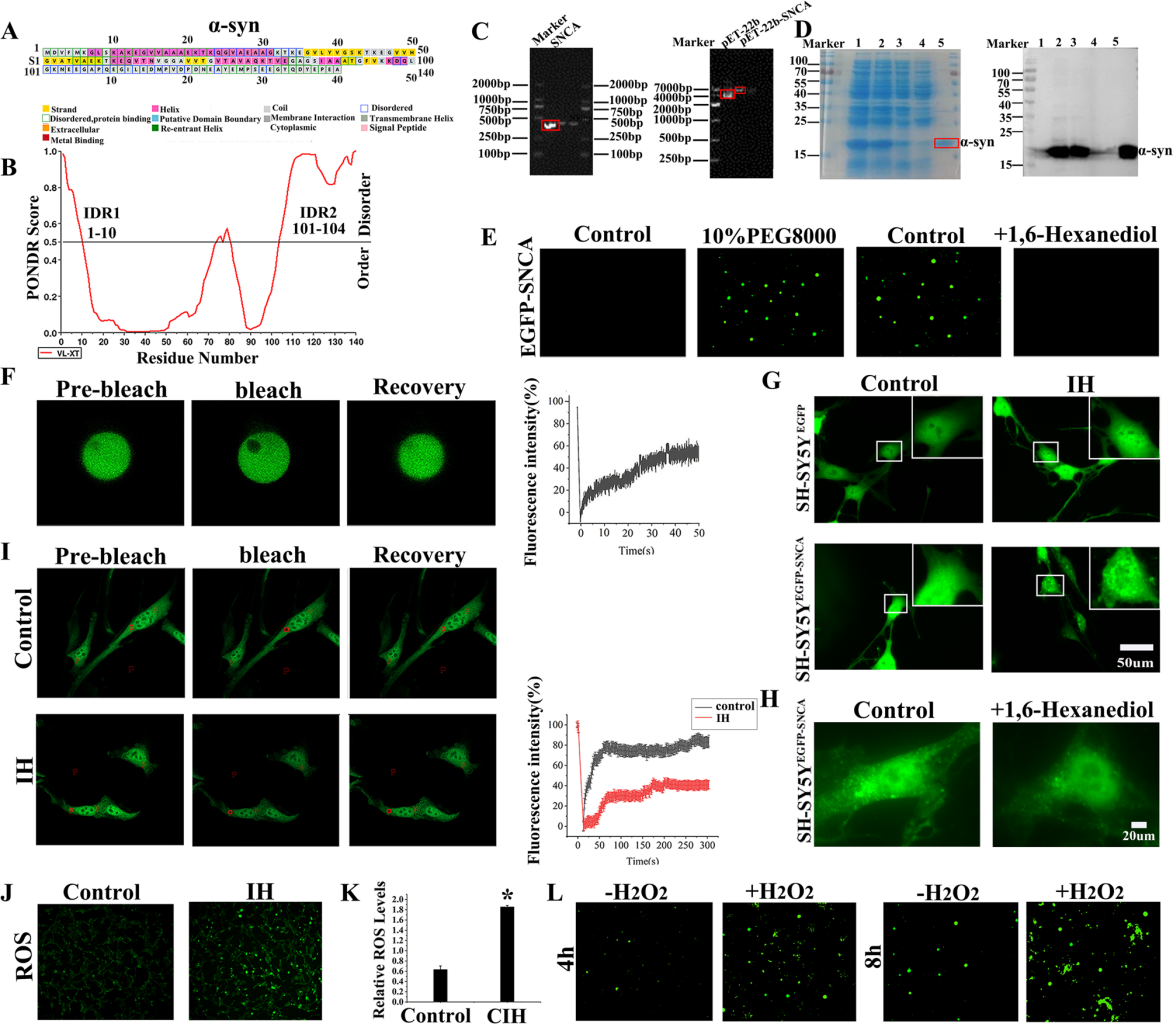
LLPS and abnormal aggregation of α-Syn. **A** The sequence plot of α-syn. **B** Prediction of disorder regions in α-syn, regions with PONDR scores exceeding 0.5 were classified as disordered, whereas those below 0.5 were designated as ordered. **C** SNCA and pET-22b double enzyme digestion results were shown by agarose gel electrophoresis, colony PCR results after ligation of pET-22b, and SNCA fragments. **D** SDS-PAGE results of α-syn purified using Ni affinity column chromatography. **E** Confocal microscopy images showed α-syn droplets formed under 10% PEG8000. Adding 10% 1,6-hexanediol to the system dissolved these droplets. **F** Images of α-syn droplet mobility measured by FRAP at different times and fluorescence bleaching recovery curves. **G** IH induced the formation of α-syn droplets and irregular aggregates in SH-SY5Y^EGFP−SNCA^ cells. **H** The addition of 10% 1,6-hexanediol to SH-SY5Y^EGFP−SNCA^ cells eliminated α-syn droplets and reduced α-syn aggregates. **I** The results depicted the FRAP of α-syn in SH-SY5Y cells expressing EGFP-SNCA, as illustrated by the fluorescence recovery curves. **J** The DCFH-DA fluorescent probe was utilized to reveal the ROS levels in SH-SY5Y cells. **K** The ROS levels in the VTA were measured. **L** Confocal microscopy images of α-syn incubated for 4 h and 8 h at room temperature, with or without H_2_O_2_. Data were presented as mean ± SD. *P < 0.05 vs. Control group

Under IH conditions, SH-SY5Y cells transfected with EGFP-SNCA exhibited numerous α-syn droplets and irregular aggregates. In contrast, under RA conditions, no obvious droplet formation was observed, and the EGFP signal remained diffuse, confirming that IH induced abnormal phase separation and aggregation of α-syn in SH-SY5Y cells (Fig. 5G). The addition of 10% 1,6-hexanediol to IH-treated SH-SY5Y ^EGFP−SNCA^ cells dissolved droplets and reduced α-syn aggregates (Fig. 5H). Intracellular FRAP experiments showed that fluorescence recovery of α-syn droplets in IH-treated cells reduced, with lower fluorescence values and decreased droplet fluidity (Fig. 5I). These results demonstrated that IH induced abnormal phase separation of α-syn, leading to solid-like phase transitions and aggregation, which impeded fluorescence recovery.

### ROS generation increased after CIH/IH exposure, and the LLPS of α-Syn was regulated by H₂O₂

Numerous factors regulate phase separation, and ROS is the crucial regulatory factor. To examine the changes in ROS levels after CIH/IH exposure, we employed the DCFH-DA probe and Oroboros Oxygraph-2 K respirometer. ROS fluorescence intensity increased in SH-SY5Y cells under IH conditions (Fig. 5J). Moreover, statistical analysis revealed that the ROS content in the CIH group was approximately 3-fold higher than that in the Control group (Fig. 5K, *P* < 0.05). These results indicated that CIH/IH exposure increased ROS levels. To examine the impact of ROS on α-syn phase separation, we introduced 20 µM H₂O₂ into the α-syn phase separation system for incubation periods of 4 h and 8 h. Confocal microscopy revealed that, with prolonged incubation times, the number and volume of α-syn droplets increased following H₂O₂ treatment, leading to droplet solidification and irregular aggregation (Fig. 5L). These findings indicated that ROS promoted α-syn phase separation and aberrant aggregation, further supporting the role of ROS in regulating α-syn LLPS under CIH/IH exposure.

### DME reduced the level of ROS and ameliorated α-syn phase separation in SH-SY5Y cells treated with IH

**Fig. 6:**
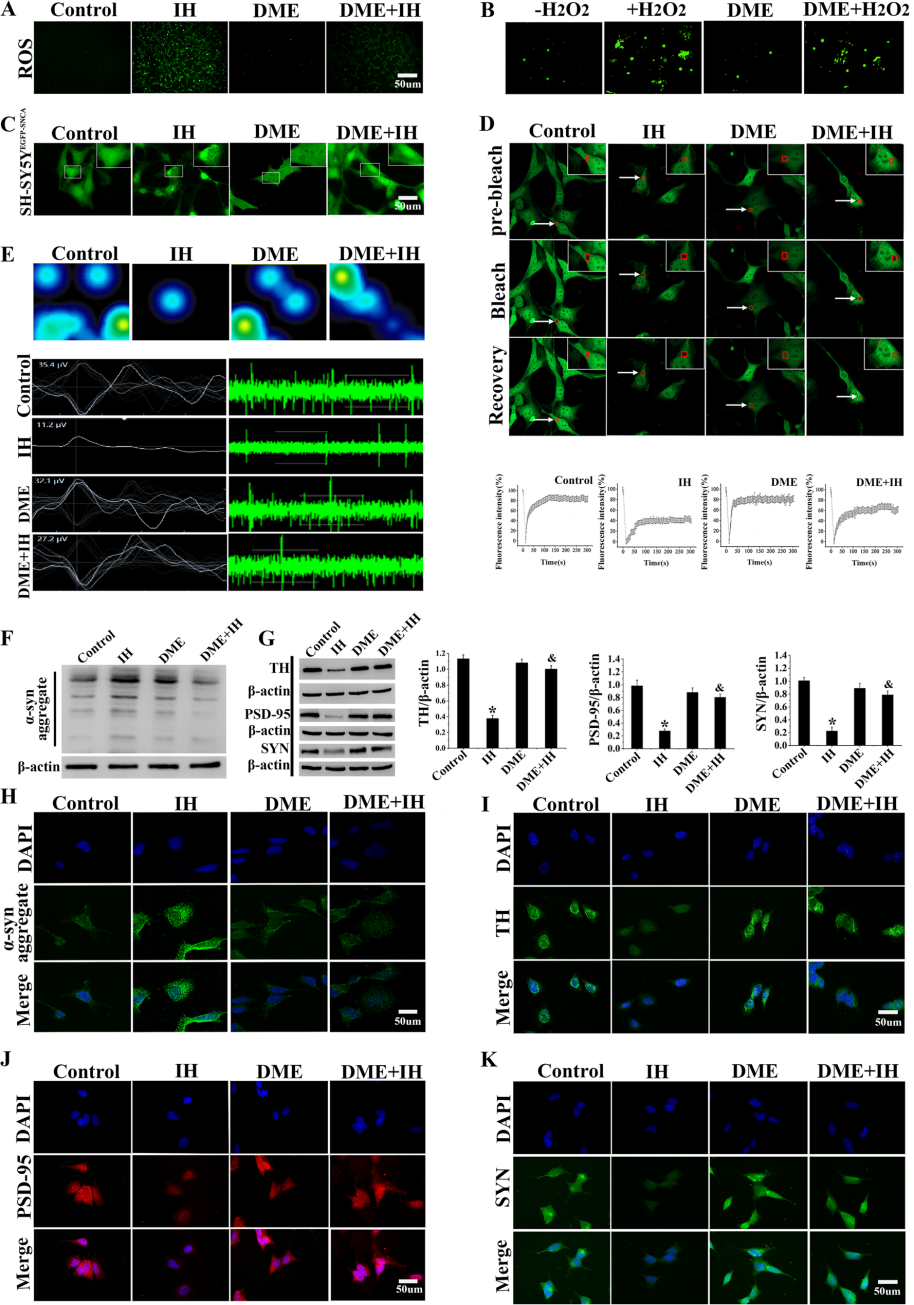
DME alleviated cell damage induced by IH. **A** ROS fluorescence intensity of SH-SY5Y cells under different treatment conditions was detected by the DCFH-DA probe. **B** Confocal microscopy images showed that DME inhibited H_2_O_2_-induced α-syn phase separation. **C** Confocal images of α-syn droplets in SH-SY5Y^EGFP−SNCA^ cells. **D** Dynamic changes in α-syn droplets under various treatments. White arrows indicate photobleaching areas, and the curves show fluorescence recovery. **E** MEA showed that DME in the DME + IH group alleviated the injury-induced decrease in SH-SY5Y cell activity compared with the IH group. **F** The expression level of α-syn aggregates was detected by Western blot. **G** Western blot and gray-value analysis of TH, PSD-95, and SYN. **H**-**K** Immunofluorescence staining images of TH, PSD-95, SYN, and α-syn aggregates in SH-SY5Y cells. Data were presented as mean ± SD. *P < 0.05 vs. Control group, ^&^*P* < 0.05 vs. IH group

Due to its potent antioxidant properties, DME was used to treat IH-exposed SH-SY5Y cells. The ROS fluorescence intensity in the DME + IH group, as detected by the DCFH-DA probe, obviously decreased (Fig. 6A). When DME and H₂O₂ were introduced into an in vitro α-syn phase separation system, DME markedly inhibited H₂O₂-induced α-syn phase separation. Specifically, the formation of α-syn droplets and aggregates was significantly reduced (Fig. 6B). Further validating the inhibitory effect of DME on α-syn phase separation in IH-treated SH-SY5Y cells, confocal microscopy revealed that DME significantly diminished α-syn droplets and irregular aggregates within neuronal cells (Fig. 6C). Additionally, FRAP experiments demonstrated that the fluorescence intensity of α-syn droplets in SH-SY5Y cells recovered to approximately 80% of the initial value within 5 min post-bleaching when treated with DME, indicating high fluidity of these droplets (Fig. 6D). These findings suggested that DME effectively suppressed abnormal α-syn phase separation in IH-treated SH-SY5Y cells, thereby reducing the formation of α-syn aggregates.

### DME mitigated the abnormal α-syn aggregation caused by IH, and relieved the SH-SY5Y cells damage

It remained unclear whether DME could mitigate neurological damage and cognitive impairment caused by abnormal aggregation of α-syn under CIH/IH conditions. MEA analysis revealed that, compared to the IH group, the neuronal action potential number, frequency, and amplitude in the DME + IH group were significantly increased, indicating that DME alleviated electrophysiological abnormalities (Fig. 6E). The results from Western blot analysis indicated that in the DME + IH group, α-syn aggregation was decreased, while expression of TH, PSD-95, and SYN proteins was increased. Specifically, the DME + IH group showed approximately 2-fold, 3-fold, and 3-fold higher levels of TH, PSD-95, and SYN, respectively, than IH group. Immunofluorescence staining also revealed enhanced expression of these proteins and reduced fluorescence intensity of α-syn in the DME + IH group (Fig. 6F-K, *P* < 0.05). These findings suggested that DME mitigated IH-induced neuronal injury by reducing abnormal aggregation of α-syn.

### DME improved the abnormal α-syn aggregation and alleviated the dopaminergic neuron damage and fear memory impairment caused by CIH

**Fig. 7:**
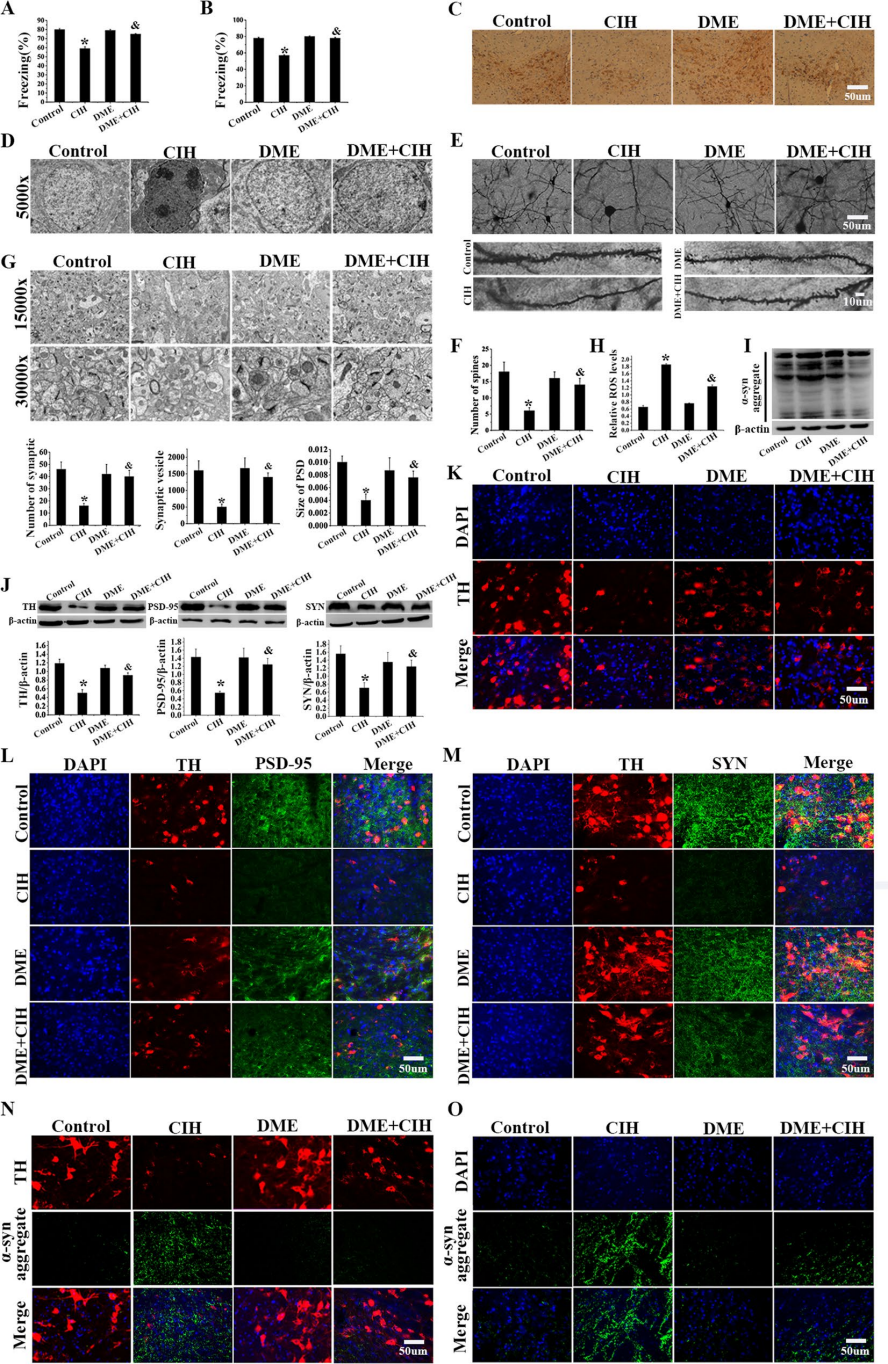
DME alleviated nerve damage and fear memory impairment induced by CIH. **A** Percentage of freezing in contextual memory test (*n* = 16). **B** Percentage of freezing in the cued memory test (*n* = 16). **C** Immunohistochemical staining results of TH-positive neurons in the VTA. **D** TEM results of neurons in the VTA. **E**-**F** Imaging and quantification of Golgi-stained neurons. **G** Electron microscopy of synapses results under different treatments, and Statistical analysis of synapse number, synaptic vesicle number, and PSD area. **H** The ROS content in the VTA of mice was detected. **I** The expression level of α-syn aggregates was detected by Western blot. **J** Western blot and gray-value analysis of TH, PSD-95, and SYN. **K**-**O** Immunofluorescence staining images of TH, PSD-95, SYN, and α-syn aggregates in the VTA. Data were presented as mean ± SD. *P < 0.05 vs. Control group, ^&^*P* < 0.05 vs. CIH group

**Fig. 8:**
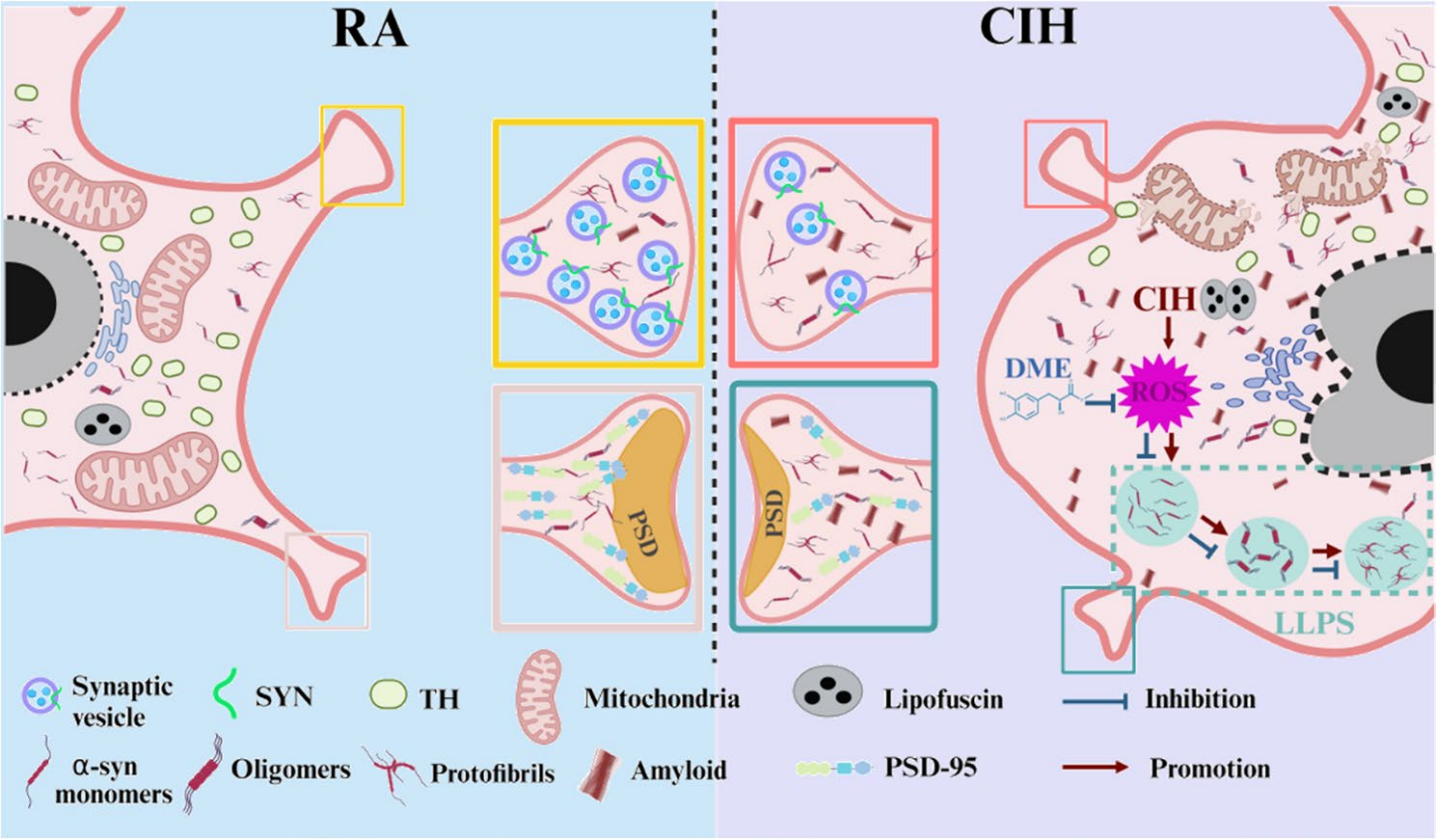
The molecular mechanism of damage in dopaminergic neurons induced by CIH/IH. Due to the highly increased ROS levels, α-syn underwent abnormal LLPS. Soluble functional α-syn monomers transform into cytotoxic oligomers and even fibril aggregates. Meanwhile, we observed pathological changes in neurons under CIH/IH conditions, including cytoplasmic vacuolation, lipofuscin accumulation, organelle damage, and synaptic injury. The antioxidant monomer DME reduced ROS production, affected abnormal α-syn LLPS and pathological aggregation, then alleviated the neuronal damage under CIH/IH exposure

To further explore the in vivo effects of DME, we conducted a series of in-depth animal studies. Contextual and cued memory assessments indicated an elevation in freezing behavior percentages in DME + CIH group compared to CIH group. The percentage increased by approximately 10% and 20% in the two types of memory tests, respectively (Fig. 7A, B, *P* < 0.05). These findings suggested that DME mitigated CIH-induced memory impairments. Subsequently, immunohistochemical analysis revealed an increase in the number of TH-positive neurons in the VTA of DME + CIH group relative to the CIH group (Fig. 7C). TEM observations indicated that DME treatment alleviated CIH-induced severe nuclear deformation, cytoplasmic vacuolization, and lipofuscin accumulation in mice VTA dopamine neurons (Fig. 7D). There was a notable increase in synapse and synaptic vesicle counts in the DME + CIH group, with clearer synaptic clefts and an enlarged PSD area. The synapse counts, synaptic vesicle counts and PSD area were approximately 2-fold, 3-fold, and 2-fold higher in the DME + CIH group than in the CIH group (Fig. 7G). Golgi staining demonstrated that the DME + CIH group exhibited a nearly 2-fold increase in dendritic spine numbers compared to the CIH group (Fig. 7E, F). Additionally, ROS expression levels were reduced in the VTA following DME treatment. ROS levels of the DME + CIH group were reduced by about 30% compared with the CIH group (Fig. 7H, *P* < 0.05). It was verified that DME mitigated α-syn aggregation and notably enhanced the expression levels of TH, PSD-95, and SYN. The expression levels of TH, PSD-95, and SYN in the DME + CIH group were approximately 2-fold, 2-fold, and 2-fold greater than those in the CIH group (Fig. 7I-O, *P* < 0.05). Collectively, results indicated that DME mitigated α-syn accumulation and thereby reduced damage to VTA dopaminergic neurons induced by CIH (Fig. 8).

## Discussion

The association between abnormal aggregation of α-syn and nerve damage as well as cognitive dysfunction has been extensively documented in various α-synucleinopathies, including Parkinson’s disease (PD) (Calabresi et al. 2023). However, what is the role of abnormal deposition of α-syn in OSA patients’ nerve damage?

Previous clinical studies have confirmed that there are nerve injuries in the hippocampus and cortex of moderate-severe OSAHS patients, which results in cognitive dysfunction (Zhou et al. 2020). In 2004, Shun Yu et al. discovered in animal experiments that repeated hypoxia-reoxygenation could induce abnormal aggregation of α-syn in cortical neurons (Shun et al. 2004). Subsequently, it was observed that total α-syn and its phosphorylated forms were significantly elevated in OSA patients’ plasma (Sun et al. 2019). Then Jixunming’s team pointed out that, hypoxia-induced abnormal aggregation of α-syn contributed to cognitive dysfunction in cerebral anoxic diseases (Li et al. 2022). Our data revealed that α-syn aggregated abnormally in neurons after CIH exposure, and the aggregation was intimately related to cognitive dysfunction. The above experiments have suggested that cognitive impairment in OSA patients is closely associated with abnormal aggregation of α-syn, but the underlying molecular mechanisms remain incompletely elucidated.

In the current study, the 10-week CIH exposure resulted in severe impairment of fear memory in mice. Classical studies confirmed that fear memory formation was related to the hippocampus, amygdala, and cortex (Marek et al. 2013; Maren and Quirk 2004). Recent research has further reported that the ventral tegmental area (VTA) is also involved in fear memory formation (Tsetsenis et al. 2023; Blaess and Krabbe 2023). After CIH exposure, mice presented with a significant reduction in freezing behavior percentages. Meanwhile, α-syn was abnormally deposited in dopamine neurons (DA), the predominant cell type of VTA. These neurons exhibited severe pathological damage, including impairment of organelles and subcellular structures, particularly at the synaptic level. It is generally known that synaptic damage is intimately linked to the formation and function of neural circuits (Südhof 2021). Some articles reported that the neural circuit from VTA-DA neurons to hippocampus CA1 was involved in fear memory formation (Adeniyi et al. 2020). Our laboratory’s previous findings also indicated that the neural projections were significantly diminished under CIH conditions. By down-regulating α-syn expression to decrease the deposition in VTA-DA neurons of CIH mice model, we observed that neuronal damage was mitigated and fear memory impairment was also ameliorated (data not shown). These results suggested that reduced neural projections were closely linked to neuronal damage caused by α-syn aggregation in VTA-DA neurons. Numerous studies confirmed that α-syn aggregation in DA neurons could result in functional impairment, which was intricately related to the onset and progression of neurodegenerative diseases like PD, particularly regarding cognitive dysfunction severity (Ganjam et al. 2019). Although α-syn deposition in VTA-DA neurons is not a primary pathological feature of PD, it does participate in the pathogenesis and significantly impacts cognitive function (Alberico 2015). For the first time, it was confirmed that abnormal α-syn aggregation in VTA-DA neurons contributed to CIH-induced fear memory impairment. To define the underlying molecular mechanisms, we further focused on the abnormal deposition of α-syn within damaged DA neurons.

There are varying views on the pathomechanism of nerve injury and cognitive impairment induced by α-syn. Some researchers posited that the cytotoxic effects of α-syn oligomers and deposition played a crucial role (Jeon et al. 2020; Forloni 2023). Others argued that the reduction of soluble functional α-syn within the cytoplasm might be the major cause (Bridi and Hirth 2018). However, we contend that neither view should be examined in isolation. Previous studies have shown that merely counteracting the abnormal deposition of α-syn is insufficient to effectively mitigate nerve damage and dysfunction (Rodger et al. 2023). Moreover, the reduction of soluble functional α-syn in the cytoplasm is closely associated with the pathological aggregation of α-syn (Zhang et al. 2023). In fact, the transition of α-syn from a functional state to abnormal deposition is an inseparable and dynamic process. The aggregation of α-syn is driven by a nucleation-dependent polymerization mechanism. Initially, monomers progressively form oligomers and fibrils, ultimately leading to amyloid aggregation (Estaun-Panzano et al. 2023). Recent studies have consistently revealed that this process is intricately linked to phase separation (Siegert et al. 2021). Maji’s team first reported that the aggregation nucleation of α-syn was associated with α-syn LLPS in vitro and in cells (Ray et al. 2020). In the current work, we observed that α-syn droplets formed and underwent a liquid-to-solid phase transition to form the amyloid state. This finding further confirmed that α-syn LLPS preceded aggregation. Therefore, clarifying the LLPS process of α-syn and its upstream regulatory mechanisms is essential for preventing aberrant deposition and maintaining the levels of soluble functional α-syn. Further, this is critical for mitigating pathological damage, enhancing cognitive function, and inhibiting the progression of neurodegeneration.

Studies have indicated that the dynamics of α-syn phase separation are highly dependent on intrinsic factors and extrinsic regulators. The intrinsic functions include the structural properties of intrinsically disordered regions (IDRs) and levels of protein post-translational modifications (PTMs) (Mukhopadhyay 2020). And the regulation of the solution environment, such as pH, temperature, ion strength, and ROS, also gets involved (Poudyal et al. 2022). In the presence of constant temperature and other environmental factors, we detected changes of vital factors (PH and ROS) within the cytoplasm, before and after IH treatment. The results showed no significant change in cytoplasmic pH (data not shown). Our experiments revealed that ROS increased in SH-SY5Y cells and VTA-DA neurons. Additionally, in vitro experiments observed that α-syn formed droplets in the presence of ROS, which subsequently progressed into oligomers. These results suggested that ROS production regulated the LLPS process of α-syn after CIH exposure, leading to its abnormal aggregation. Except for cytoplasmic environment factors, the phase separation of α-syn is critically modulated by its interactions with other proteins and RNA molecules in its neural functional realization. Li Dan and colleagues found the multi-component co-phase separation of α-syn with VAMP2 and synaptic vesicles (SVs) promoted SV clustering at synapses, thereby regulating neurotransmitter release (Wang et al. 2024). It was reported that RNA G-quadruplex assembly induced α-syn’s sol-gel phase transition, which was linked to its nuclear function in gene expression regulation (Matsuo et al. 2024). Therefore, investigating these interactions can enhance our understanding of phase separation’s role in cellular physiology and disease pathogenesis. Advances in techniques like surface plasmon resonance (SPR), microcalorimetry, and PROTAC-targeted protein-LLPS regulation, offer a potential opportunity into the dynamic regulation and deposition mechanisms of α-syn LLPS (Shi et al. 2023).

ROS serve as important regulators of phase separation, and there have been numerous therapeutic approaches aimed at ROS modulation. Extensive research has shown that chemical agents such as NADPH oxidase inhibitors and ROS-specific scavengers (e.g., nitrones) can mitigate pathological damage caused by ROS (Cipriano et al. 2023). However, some chemicals face challenges in dose control. Inadequate doses might fail to effectively scavenge ROS, while excessive doses could be toxic. Besides, prolonged use might lead to significant side effects (Kavčič et al. 2019). In recent years, Chinese herbal monomers have attained increasing attention due to their remarkable potential in mitigating ROS (Zong et al. 2020). The dosing flexibility of Chinese herbal monomers offers a broader safety margin and fewer side effects (Ravipati et al. 2012). Danshensu, an effective water-soluble component of the traditional Chinese herb*Salvia miltiorrhiza*, has multiple biological properties such as anti-inflammatory and antioxidant effects (Zhao et al. 2008). However, its poor chemical stability limits its clinical application. Our laboratory’s previous research has found that SMND-309, a derivative of Danshansu, has a stable chemical structure and good antioxidant properties, and can inhibit oxidative stress induced by IH/CIH, thereby improving neurologic injury and cognitive impairment (Hou et al. 2022). To further enhance the application in the field of neural injury treatment, we developed the DME, a modified ester derivative of Danshensu. The lipophilic and small-molecule properties allow DME to penetrate the blood-brain barrier more effectively. DME also has stronger antioxidant effects and a more stable chemical structure. In vitro studies initially confirmed that DME mitigated H_2_O_2_-induced abnormal α-syn LLPS and improved α-syn nucleation abnormalities. Further cellular experiments revealed that DME reduced ROS levels, corrected the abnormal α-syn LLPS process caused by IH, inhibited α-syn droplet and aggregate formation. Meanwhile, we discovered DME indeed alleviated neuron injury and cognitive impairment in CIH-induced animal models. These findings have indicated that DME has superior neuroprotective effects.

## Conclusion

In sum, our research provides new molecular targets for treating α-syn pathological aggregation and neurodegeneration by focusing on α-syn LLPS regulation. Additionally, it lays the foundation for the potential application of DME in rescuing damaged nerve cells and improving cognitive function.

## Data Availability

No datasets were generated or analysed during the current study.

## Abbreviations

OSA: Obstructive sleep apnea
CIH: Chronic intermittent hypoxia
VTA: Ventral tegmental area
SF: Sleep fragmentation
α-syn: A-synuclein
IH: Intermittent hypoxia
LLPS: Liquid-liquid phase separation
ROS: Reactive oxygen species
DME: Danshensu methyl ester
WT: Wild-type
MEA: Microelectrode array
TEM: Transmission electron microscopy
TH: Tyrosine hydroxylase
PSD-95: Postsynaptic density protein 95
SYN: Synaptophysin
FRAP: Fluorescence recovery after photobleaching
EGFP: Enhanced green fluorescent protein
