# Correction: Molecular mechanisms of α-syn abnormal phase separation in cognitive impairment induced by chronic intermittent hypoxia and the neuroprotective effects of Danshensu methyl ester

**DOI:** 10.1186/s10020-026-01519-z

**Published:** 2026-06-08

**Authors:** Juan Li, Na Zhang, Ziyin Zhang, Jinsai Fu, Wenjing Ren, Yi Sun, Shuling Song, Xiaoqian Liu, Jinghui Liu, Jingyu Wang, Yunliang Sun, Kai Zhang, Rongrong Guo, Changjun Lv, Lei Pan, Guiwu Qu, Fang Han, Yan Yu

**Affiliations:** 1 Department of Respiratory Medicine, Binzhou Medical University Hospital, Binzhou Medical University, Binzhou, 256603 China; 2 Department of The First School of Clinical Medicine, Binzhou Medical University, Yantai, 264003 China; 3 School of Basic Medical Sciences, Binzhou Medical University, Yantai, 264003 China; 4 Department of The Second School of Clinical Medicine, Binzhou Medical University, Yantai, 264003 China; 5 School of Pharmacy, Binzhou Medical University, Yantai, 264003 China

**Correction: Molecular Medicine 31, 320 (2025)**

**https://doi.org/10.1186/s10020-025-01366-4**

In this article (Li, et al. 2025), Juan Li, Na Zhang, Ziyin Zhang, and Jinsai Fu should have been denoted as equally contributing authors and co-first authors.

The original article has been corrected.
